# Supplementary material for: Prevalence and Clinicopathological Characteristics of Moderate and High-Penetrance Genes in Non-BRCA1/2 Breast Cancer High-Risk Spanish Families
Source: J Pers Med. 2021 Jun 12;11(6):548. doi: 10.3390/jpm11060548 (PMC8231620; doi:10.3390/jpm11060548)

Supplementary Materials

Table S1. Variants of unknow signficante (VUS) in the study cohort

Gene Variants									Personal history			Family history		
Study ID	Gene	Variant type	Class[1]	HGVS coding	HGVS protein	Transcripts	rsID	2nd variant	BC age	Subtype (ER/PR/HER2)	Bilateral BC	No. cancers	No. BCs	No. OCs
17044	BRCA1	Missense	3	c.3701T>G	p.Val1234Gly	NM_07300.4	rs587778137	-	65	−/-/+	Yes	3	3	0
32917	BRCA2	Missense	3	c.6443C>A	p.Ser2148Tyr	NM_000059.4	rs80358880	-	52	+/+/-	No	3	3	0
47419	BRCA2	Missense	3	c.8182G>C	p.Val2728Leu	NM_000059.4	rs28897749	-	33	+/+/–	Yes	8	5	0
49976	PALB2	Missense	3	c.1250C>A	p.Ser417Tyr	NM_024675.4	rs45510998	-	44	+/+/+	No	5	4	0
47831	ATM	Missense	3	c.8810T>C	p.Val2937Ala	NM_000051.4	rs587782149	-	27	+/-/-	No	3	3	0
55180	ATM	Missense	3	c.7202T>C	p.Ile2401Thr	NM_000051.4	rs1555122117	-	36	+/+/–	No	3	3	0
54784	ATM	Missense	3	c.6860G>A	p.Gly2287Glu	NM_000051.4	rs1800061	-	36	+/+/–	No	3	2	1
33654	ATM	Missense	3	c.8810T>C	p.Val2937Ala	NM_000051.4	rs587782149	-	39	+/+/–	No	4	4	0
13788	ATM	Missense	3	c.2269G>A	p.Gly757Arg	NM_000051.4	rs587779819	-	49	+/+/–	No	5	4	0
60770	ATM	Missense	3	c.610G>A	p.Gly204Arg	NM_000051.4	rs147915571	BARD1 c.1916G>A (p.Arg639His) Class 3	49	+/+/–	No	6	3	3
41863	CHEK2	Missense	3	c.1036C>T	p.Arg346Cys	NM_007194.4	rs201206424	-	56	+/+/–	No	3	3	0
30613	CHEK2	Missense	3	c.1684C>G	p.Arg562Gly	NM_007194.4	-	-	36	+/+/–	No	3	3	0
58063	CHEK2	Missense	3	c.1684C>G	p.Arg562Gly	NM_007194.4	-	-	42	+/+/+	Yes	3	3	0
42973	CHEK2	Missense	3	c.1684C>G	p.Arg562Gly	NM_007194.4	-	-	56	+/+/-	Yes	5	2	2
50094	CHEK2	Missense	3	c.1733G>A	p.Arg578His	NM_007194.4	rs41295288	MEN1 c.1534G>A (p.Gly512Ser) Class 3	48	+/-/-	No	3	3	0
42603	CHEK2	Missense	3	c.1246A>G	p.Lys416GluA	NM_007194.4	rs74751600	-	61	+/+/-	No	2	2	0
41064	BRIP1	Missense	3	c.1941G>T	p.Trp647Cys	NM_032043.2	rs786202760	-	44	+/+/+	No	3	3	0
31547	BRIP1	Missense	3	c.1941G>T	p.Trp647Cys	NM_032043.2	rs786202760	-	53	-/-/-	Yes	3	3	0
45784	MSH2	Missense	3	c.1787A>G	p.Asn596Ser	NM_000251.2	rs41295288	-	44	+/+/-	No	3	3	0
36289	MSH2	Missense	3	c.2772T>G	p.Asn924Lys	NM_000251.2	-	RAD50 c.980G>A (p.Arg327His) Class 3	49	+/+/-	No	4	3	0
55832	NBN	Missense	3	c.1136C>T	p.Pro379Leu	NM_002485.5	rs367760321	-	40	+/-/–	No	3	3	0
38266[2]	NBN	Frameshift	3	c.1238A>G	p.Asn413Ser	NM_002485.5	rs529340553	CASP8 c.331delG (p.Ala111Leufs*22)Class 4	57	+/+/–	No	7	3	0

BC: breast cancer. ER: Estrogen Receptor. PR: Progesterone receptor. OC: Ovarian cancer.

Databases used: ClinVar: Clinical Variation (https://www.ncbi.nlm.nih.gov/clinvar/); VarSome: the Human Genomics Community (https://varsome.com/).

[1]Class: Variant classification proposed by the American College of Medical Genetics and Genomics (ACMG) (deleterious: class 5; likely deleterious: class 4; VUS: class 3; likely benign: class 2; and benign: class 1

Figure S1. Quality by base throughout the readings. The X axis represents the position in the reading and the Y axis the quality value of the bases on the Phred scale. A Phred value of 20 assumes 1 error in 20, an error of 30 means that there is 1 error in 1000, and successively. For each position in the readings, the distribution of quality values is analyzed and one boxplot per base is generated. The red line represents the median while the blue line represents the mean.

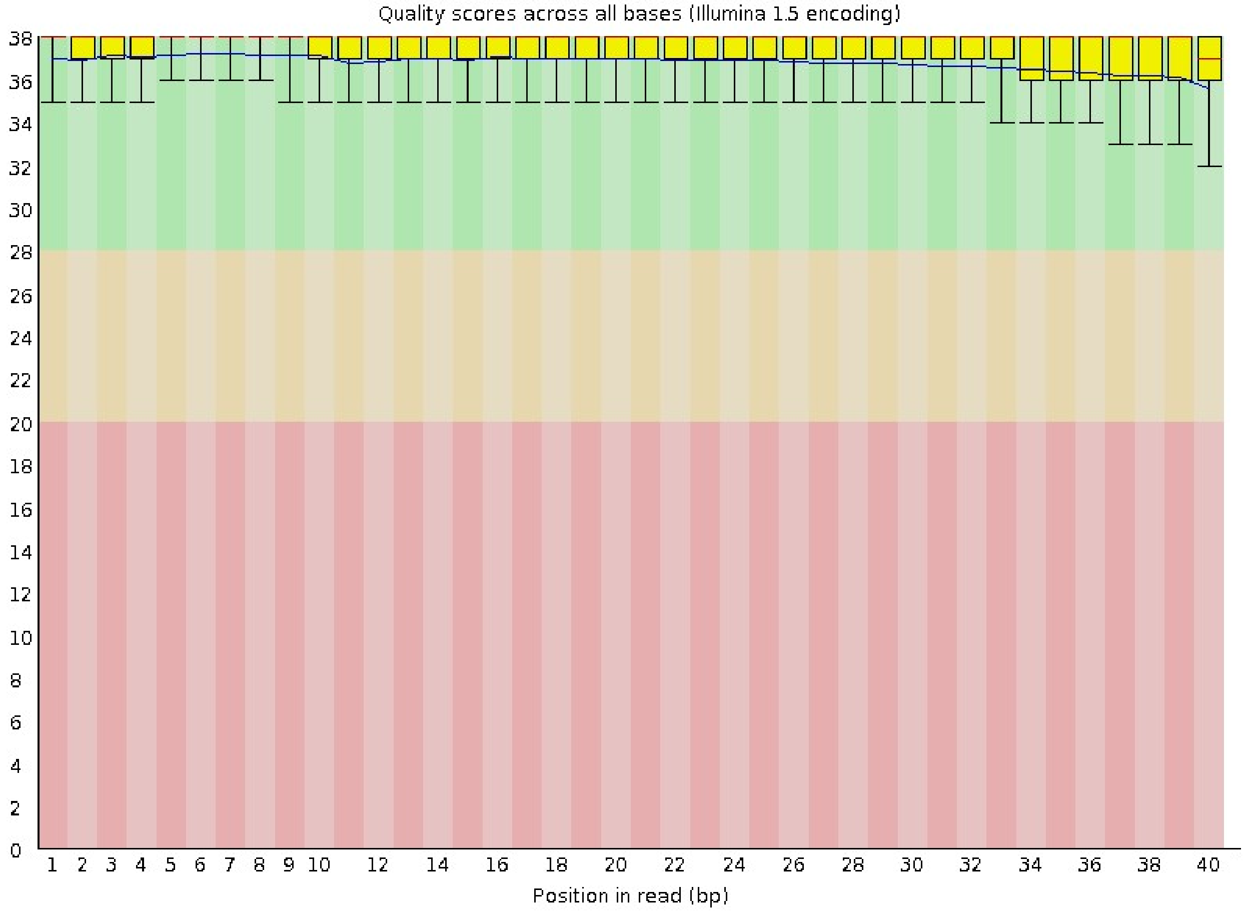

Figure S2. Quality values per read. This chart represents the average quality values per read. On the X axis it represents the quality value and on the Y axis the number of readings.

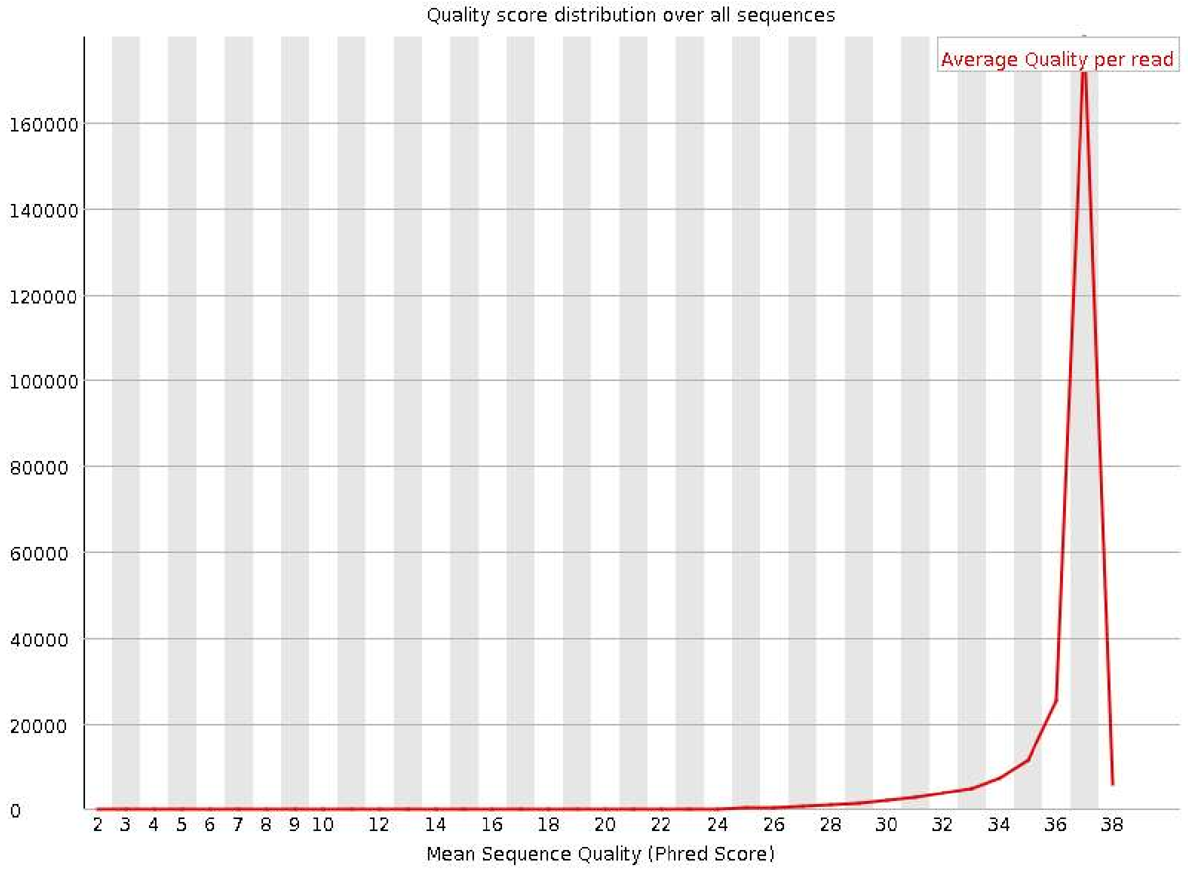

Supplement: Supplementary file 1 [file jpm-11-00548-s001.zip › jpm-1218134-supplementary.pdf]
